# A 2-Year Single-Centre Audit on Antibiotic Resistance of *Pseudomonas aeruginosa*, *Acinetobacter baumannii* and *Klebsiella pneumoniae* Strains from an Intensive Care Unit and Other Wards in a General Public Hospital in Greece

**DOI:** 10.3390/antibiotics8020062

**Published:** 2019-05-15

**Authors:** Georgios Feretzakis, Evangelos Loupelis, Aikaterini Sakagianni, Nikoletta Skarmoutsou, Sophia Michelidou, Aikaterini Velentza, Maria Martsoukou, Konstantinos Valakis, Stavroula Petropoulou, Emmanouil Koutalas

**Affiliations:** 1 School of Science and Technology, Hellenic Open University, 26335 Patras, Greece; 2 IT Department, Sismanogleio General Hospital, 15126 Marousi, Greece; v_loupelis@sismanoglio.gr (E.L.); spetrop@sismanoglio.gr (S.P.); 3 Department of Quality Control, Research and Continuing Education, Sismanogleio General Hospital, 15126 Marousi, Greece; 4 Intensive Care Unit, Sismanogleio General Hospital, 15126 Marousi, Greece; sakagianni@sismanoglio.gr (A.S.); sofimicheli@yahoo.gr (S.M.); valakis@sismanoglio.gr (K.V.); 5 Microbiology Laboratory, Sismanogleio General Hospital, 15126 Marousi, Greece; lettaskarm@yahoo.gr (N.S.); micr.fym@sismanoglio.gr (A.V.); mikroviologiko@sismanoglio.gr (M.M.); 6 Administration, Sismanogleio General Hospital, 15126 Marousi, Greece; dioikitis@sismanoglio.gr

**Keywords:** antibiotic resistance, antimicrobial resistance, *Pseudomonas aeruginosa*, *Acinetobacter baumannii*, *Klebsiella pneumoniae*, intensive care unit

## Abstract

Hospital-acquired infections, particularly in the critical care setting, are becoming increasingly common during the last decade, with Gram-negative bacterial infections presenting the highest incidence among them. Multi-drug-resistant (MDR) Gram-negative infections are associated with high morbidity and mortality, with significant direct and indirect costs resulting from long hospitalization due to antibiotic failure. As treatment options become limited, antimicrobial stewardship programs aim to optimize the appropriate use of currently available antimicrobial agents and decrease hospital costs. *Pseudomonas aeruginosa*, *Acinetobacter baumannii* and *Klebsiella pneumoniae* are the most common resistant bacteria encountered in intensive care units (ICUs) and other wards. To establish preventive measures, it is important to know the prevalence of Gram-negative isolated bacteria and antibiotic resistance profiles in each ward separately, compared with ICUs. In our single centre study, we compared the resistance levels per antibiotic of *P. aeruginosa*, *A. baumannii* and *K.pneumoniae* clinical strains between the ICU and other facilities during a 2-year period in one of the largest public tertiary hospitals in Greece. The analysis revealed a statistically significant higher antibiotic resistance of the three bacteria in the ICU isolates compared with those from other wards. ICU strains of *P. aeruginosa* presented the highest resistance rates to gentamycin (57.97%) and cefepime (56.67%), followed by fluoroquinolones (55.11%) and carbapenems (55.02%), while a sensitivity rate of 97.41% was reported to colistin. A high resistance rate of over 80% of *A. baumannii* isolates to most classes of antibiotics was identified in both the ICU environment and regular wards, with the lowest resistance rates reported to colistin (53.37% in ICU versus an average value of 31.40% in the wards). Statistically significant higher levels of resistance to most antibiotics were noted in ICU isolates of *K. pneumoniae* compared with non-ICU isolates, with the highest difference—up to 48.86%—reported to carbapenems. The maximum overall antibiotic resistance in our ICU was reported for *Acinetobacter* spp. (93.00%), followed by *Klebsiella* spp. (72.30%) and *Pseudomonas* spp. (49.03%).

## 1. Introduction

Rapid dissemination of antibiotic-resistant Gram-negative infections constitute a worldwide problem with an increasing health and economic burden [1]. A recently published European Centre for Disease Prevention and Control (ECDC) study estimates that about 33,000 people die each year as a direct consequence of an infection due to bacteria resistant to antibiotics [2]. Healthcare associated infections (HAI’s) account for the major burden of these multidrug-resistant infections, while last-line treatments, such as carbapenems and colistin, become increasingly less reliable, limiting the available therapeutic options [3].

According to the European Antimicrobial Resistance Surveillance Network (EARS-Net) data from 2015, Greece is among the countries with the greatest burden of infections due to antibiotic-resistant bacteria in the EU and EEA [4]. In 2015, most of this burden was due to infections with carbapenem-resistant or colistin-resistant bacteria [2,4,5]. A mean incidence of 0.48 per 1000 patient-days and a crude 28-day mortality rate of 34.4% were reported from the Hellenic Center for Disease Control and Prevention (HCDCP) in 2014, in the context of a national action plan to combat carbapenem-resistant Gram-negative pathogens in acute-care hospitals in Greece [6].

Moreover, the annual report of the EARS-Net for 2017 shows that Greece has the highest percentage of resistance to carbapenems and fluoroquinolones for *Klebsiella pneumoniae* among all European countries, with a combined resistance to three or more antimicrobial groups among piperacillin/tazobactam, ceftazidime, fluoroquinolones, aminoglycosides and carbapenems being above 30% for *Pseudomonas aeruginosa*, with resistance of *Acinetobacter baumannii* to carbapenems being raised to 94% [7].

Antimicrobial stewardship programs aim to optimize the appropriate use of currently available antimicrobial agents to improve therapeutic results for Gram-negative multi-drug-resistant (MDR) infections, reducing antimicrobial resistance and decreasing hospital costs [8]. *P. aeruginosa*, *A. baumannii*, and *K. pneumoniae* are encountered more frequently than other pathogens in intensive care units (ICUs). To establish preventive measures, it is crucial to know the antibiotic resistance profiles in each ward separately compared with ICUs. The aim of the present study is to compare the resistance levels of *P. aeruginosa*, *A. baumannii* and *K. pneumoniae* isolates between ICUs and other facilities in two consecutive years, from January 2017 until December 2018, in one of the largest public tertiary hospitals in Greece. This will help to implement more effective strategies for the reduction of multidrug resistance.

## 2. Results

### 2.1. Statistical Analysis for Pseudomonas aeruginosa

We analyzed the overall 2-year and semester resistance rates in 21,836 tests of *P. aeruginosa* against routinely tested individual antimicrobial agents (Table A1). All analyses was performed with SPSS Statistics version 24.0 (IBM, Armonk, NY, USA) [9]. The relevant data were analyzed in a two year period (2017–2018) and each semester was analyzed separately. Differences between means regarding the resistance of *P. aeruginosa* between ICUs and the wards (Internal Medicine A, Pulmonary A, Internal Medicine B, Pulmonary B, Cystic Fibrosis Unit, Urology) were determined by univariate analysis of variance and Tukey’s multiple range tests. A significance level of 0.05 was used. The test results are given in the corresponding tables in the appendix (Table A2 and Table A3).

### 2.2. Statistical Analysis for Acinetobacter baumannii

We analyzed the overall 2-year and semester resistance rates in 5609 tests of *A. baumannii* against routinely tested individual antimicrobial agents (Table A4). The relevant data were analyzed in a two year period (2017–2018) and each semester separately. A significance level of *p* < 0.05 was used. Differences between means regarding the resistance of *A. baumannii* between ICUs and the wards (Internal Medicine A, Pulmonary A, Pulmonary B) were determined by univariate analysis of variance and Tukey’s multiple range tests. The test results are given in the corresponding tables (Table A5 and Table A6).

### 2.3. Statistical Analysis for Klebsiella pneumoniae

We analyzed the overall 2-year and semester resistance rates in 14,918 tests of *K. pneumoniae* against routinely tested individual antimicrobial agents (Table A7). The relevant data were analyzed in a two year period (2017–2018) and in each semester separately. Differences between means regarding the resistance of *K. pneumoniae* between ICUs and the wards (Internal Medicine A, Pulmonary A, Internal Medicine B, Pulmonary B, Urology) were determined by univariate analysis of variance and Tukey’s multiple range tests. A significance level of *p* < 0.05 was used. The test results are given in the corresponding tables (Table A8 and Table A9).

## 3. Discussion

Despite significant advances in the intensive care setting during the last decade, healthcare-associated infections are a potentially modifiable source of morbidity and mortality. The prevalence of ICU-acquired infections remains significantly higher than those encountered among non-ICU patients [10]. Several factors, such as a greater severity of illness, underlying conditions, immunosuppression or impaired host responses, exposure to multiple invasive devices and procedures and increased patient contact with healthcare personnel in a small specialized area may contribute to the increased risk of infection in ICU patients [11,12]. Invasive devices and procedures, such as endotracheal intubation and urinary and central venous catheters, facilitate the entry of infectious organisms into the body. The proportion of healthcare-associated infections caused by MDR Gram-negative bacteria (GNB) is on the rise, and as treatment options are scarce, it must be a global priority of the healthcare community to monitor the prevalence and spread of antimicrobial resistance [1,2,3].

The aim of the present study was to survey the overall resistance rates in ICUs compared with the non-ICU environment of our hospital, with regard to the three most commonly encountered MDR pathogens. A significantly worse antibiotic resistance profile in all three examined bacteria was observed in the ICU setting throughout the study period. Another survey [13] on the prevalence of multidrug resistance of GNB isolates in ICUs in United States and European hospitals compared with non-ICU wards from 2009 to 2011 suggested that overall resistance rates were generally higher among ICU isolates compared with non-ICU isolates. Among these three pathogens, the most frequent in our ICU were *P. aeruginosa* (224), followed by *K. pneumoniae* (204) and *A. baumanii* (195), as shown in Table A1, Table A4 and Table A7.

**Figure 1:**
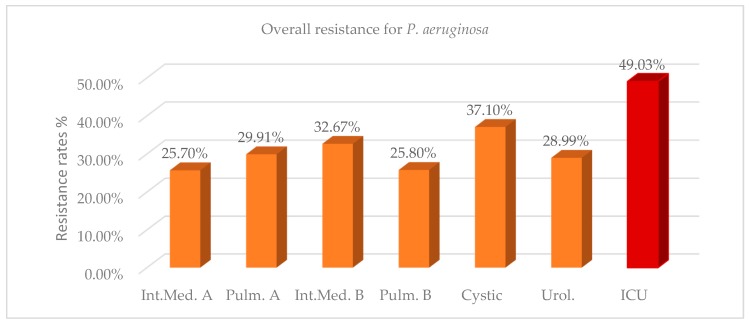
Resistance rates for *Pseudomonas aeruginosa* per ward during the 2-year period (2017–2018).

The overall resistance of *P. aeruginosa* in the ICU during the two years 2017–2018 was 49.03%, while in the other wards resistance ranged from 25.70% to 37.10% (Figure 1, Table A1). Mean differences of resistance between ICU and non-ICU isolates during the two years were statistically significant and ranged from 11.93% to 23.33%. The number of isolates and resistance rate increased in the ICU in 2018 compared with 2017, while there was a corresponding reduction in the wards, except in the cystic fibrosis unit, where both values remained approximately in the same levels (Table A1). The high antibiotic resistance rate against *P. aeruginosa* strains (37.10%), as well as the high prevalence of this pathogen (46% of total isolates) in the cystic fibrosis unit can be explained by the distinct microbiota and the predominance of the pathogen in the airways of these patients [14]. As shown in Table A2, more than 47% of the ICU strains were resistant to any tested antibiotic, except colistin, where a sensitivity of 97.41% was reported. We can also observe that ICU strains present the highest resistance rates to gentamycin (57.97%) and extended-spectrum cephalosporins (56.67%), followed by fluoroquinolones (55.11%) and carbapenems (54.76–55.02%). These rates were significantly higher than those observed in most of the wards (Table A3). The high percentage of *P. aeruginosa* resistance to aminoglycosides (41.88%) and ciprofloxacin (46.22%) encountered in the urology department might be attributed to the increased in-and-out-of-hospital usage of these antibiotics for the treatment of urinary tract infections. An emerging *P. aeruginosa* resistance to colistin in up to 6% of the isolates can also be observed in the cystic fibrosis unit. High levels of resistance to aztreonam in both ICU and non-ICU strains of *P. aeruginosa* were detected, ranging from 38.21 to 46.22% (Table A2).

**Figure 2:**
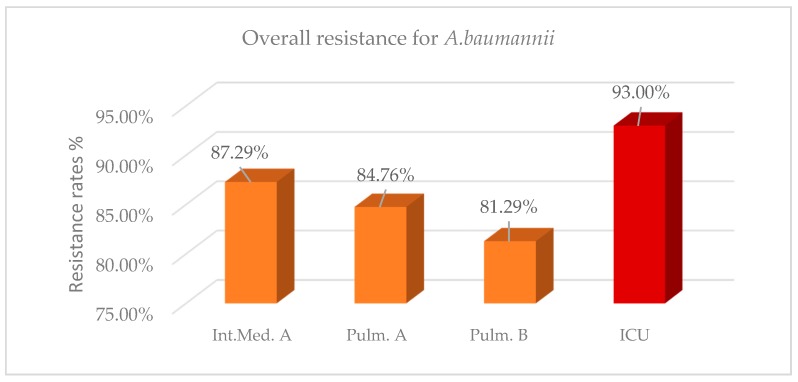
Resistance rates for *Acinetobacter baumannii* per ward during the 2-year period (2017–2018).

For the same 2-year period, overall resistance of *A. baumanii* in the ICU was 93% (Figure 2, Table A4). A high resistance rate above 80% was also identified in the other three wards; however, it was significantly lower compared with the ICU in the three last semesters, while in the first semester a nonsignificant difference was reported. As shown in Table A4, mean differences of resistance between ICU isolates and non-ICU isolates were statistically significant and ranged from 5.71% to 11.72%. In 2018, a decrease in the number of isolates with a slight decrease in the resistance rates was observed in the wards, while there was an increase in both the number of isolates and resistance rate in the ICU. Recent studies [15,16,17] have also shown a high prevalence of *A. baumanii*, ranging from 87% to 95.9%. As shown in Table A5, both ICU isolates and isolates from the wards of *A. baumannii* present an extremely high resistance to most antibiotics over 80%, with the lowest percentage reported to colistin. Colistin resistance in the ICU was 53.37%, while in the wards was on average 31.40%. Comparing the resistance of *A. baumannii* to specific antibiotics between ICU and non-ICU isolates, the highest mean difference of resistance was observed to colistin (up to 27.23%). On the other hand, no significant difference concerning *A. baumannii* resistance to ampicillin/sulbactam, trimethoprim/sulfomethoxazole (TMP/SMX), and minocycline was found between ICU isolates and isolates from the wards (Table A6).

**Figure 3:**
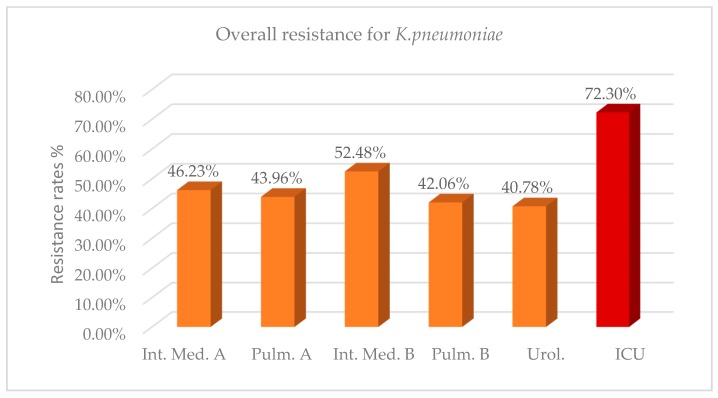
Resistance rates for *Klebsiella pneumoniae* per ward during the 2-year period (2017–2018).

Regarding *K. pneumoniae* strains, overall 2-year resistance in the ICU environment was on average 72.30% (Figure 3, Table A7). This rate was significantly higher than the resistance rate observed in the other wards, with mean differences ranging from 19.82% to 31.52% in the 2 years (Table A7). As it concerns the number of isolates, in 2018 compared with 2017, there was a significant decrease in the wards, except for ICUs and the urology department, where an increase was reported. In 2018, a significant reduction on the antibiotic resistance of *K. pneumoniae* isolates was captured in both the ICU and non-ICU environments, except in the urology department, where there was a slight increase. This fact could be attributed to the effectiveness of the surveillance measures implemented in our hospital or the antibiotic policy. A further long-term survey of the resistance profile is required to extract safe conclusions. Regarding the resistance of ICU isolates of *K. pneumoniae* to specific classes of antibiotics, the highest resistance rate was reported to older beta lactams/beta lactamase inhibitors (amp/sulb 85.98%), fluoroquinolones (79.04% to 83.04%), carbapenems (78.31% to 81.44%) and third generation cephalosporins (80.21% to 81.61%). Less than 40% of ICU isolates are resistant to colistin, versus an average rate of 13.83% reported in non-ICU isolates (Table A8). The low resistance rate of ICU isolates to tigecycline (3.57%) makes it a suitable antibiotic option for the treatment of certain *K. pneumoniae* infections. Statistically significant higher levels of resistance to most classes of antibiotics were noted in ICU isolates of *K. pneumoniae* compared with isolates from the wards, with the highest difference reported to carbapenems at up to 48.86% (Table A8 and Table A9).

In our study, the maximum overall antibiotic resistance in ICUs was reported for *Acinetobacter* spp. (93.00%), followed by *Klebsiella* spp. (72.30%) and *Pseudomonas* spp. (49.03%). In a similar study [18], the antimicrobial resistance rates were 82.10%, 52.40%, and 56.90%, respectively.

## 4. Materials and Methods

This is a cross-sectional study conducted in a public tertiary hospital in Greece, based on data from the Microbiology Department.

### 4.1. Sample-Source of Isolates

During the 2-year period (January 2017 to December 2018) a total of 888 clinical samples from 345 ICU patients and 5086 samples from 4090 patients hospitalized in other wards were included in this study and proceeded by the Microbiology Laboratory according to established protocols [19,20,21]. The samples included blood, tracheobronchial aspirates, bronchoalveolar lavage fluid, urine, skin, wounds and soft tissue specimens, intravascular catheters and pleural and peritoneal fluid. Blood cultures were incubated in the BacT/Alert system (bioMerieux, Marcy-l’Étoile, France). Isolation and identification of pathogens were carried out according to classical microbiological procedures [22].

### 4.2. Antimicrobial Susceptibility Data

Antimicrobial susceptibility testing was performed by the MicroScan system (Siemens, Munich, Germany), according to CLSI guidelines [23,24] and the results were confirmed, when necessary, using a gradient MIC (minimum inhibitory concentration) determining method following the manufacturer’s guidelines (e.g., the E-test bioMerieux, Solna, Sweden). The MICs of colistin were retested via microtiter plates (SensiTestColistin, Liofilchem, Roseto degli Abruzzi, Italy). Sensitivity and resistance breakpoints for the antibiotics were determined according to CLSI interpretive criteria [23,24] and for tigecycline according to Eucast criteria [25]. *Escherichia coli* ATCC 25922 strain and *Pseudomonas aeruginosa* ATCC 27853 were used as quality control strains for susceptibility testing.

The phenotypic detection of the production of extended-spectrum beta-lactamases (ESBL) was performed by the double disk synergy test (DDST) according to CLSI guidelines [23]. Metallo-beta-lactamases (MBL) and carbapenemases (KPC) were detected phenotypically by (a) the modified Hodge test [23], (b) the combined disk test, with a meropenem (MER) disk alone, a MER disk plus phenyl boronic acid (PBA), a MER disk plus EDTA and a MER disk plus PBA and EDTA (as described by Tsakris et al. [26]) and (c) the NG CARBA 5 immunochromatographic assay, targeting KPC-, NDM-, VIM- and IMP-type and OXA-48-like carbapenemases following the manufacturer’s guidelines (data presented at 29th ECCMID 2019 [27]. *P. aeruginosa* strains were tested phenotypically for MBL either by a combined disk test using imipenem (IPM) disk and IPM plus EDTA as described by Yong et al. [28] or by IPM-EDTA double-disk synergy test (DDST) performed as described by Lee et al. [29].

All strains that phenotypically produced more than one or no carbapenemases, the OXA producers and all those tested with NG CARBA 5, were subjected to PCR for *bla_NDM_*, *bla_VIM_*, *bla_KPC_*, and *bla_OXA-48_* genes. They were also examined for the presence of the plasmid-mediated *mcr*-1 gene for colistin-resistance (data presented at the 28th and 29th ECCMID 2019 [27,30]).

### 4.3. Study Design

This study included non-duplicate clinical isolates from different patients. All isolates with similar susceptibility patterns from a single patient were excluded. The resistance for *P. aeruginosa* was measured based on the following antibiotics: amikacin, aztreonam, cefepime, ceftazidime, ciprofloxacin, colistin, gentamicin, imipenem, meropenem, doripenem, piperacillin/tazobactam, tobramycin and levofloxacin. The resistance for *A. baumannii* was measured based on the following antibiotics: amikacin, ampicillin/sulbactam, cefepime, cefotaxime, ceftazidime, ciprofloxacin, colistin, gentamicin, imipenem, levofloxacin, meropenem, minocycline, tobramycin, trimethoprim/sulfamethoxazole, tetracycline and tigecycline. The resistance for *K. pneumoniae* was measured based on the following antibiotics: amikacin, amoxicillin/clavulanic acid, ampicillin/sulbactam, cefepime, cefotaxime, cefoxitin, ceftazidime, cefuroxime, ciprofloxacin, colistin, ertapenem, gentamicin, imipenem, meropenem, piperacillin/tazobactam, tetracycline, tobramycin, trimethoprim/sulfamethoxazole, levofloxacin, moxifloxacin and tigecycline.

Our research focuses only on the antibiotics mentioned above since there is an adequate number of samples for these for deducing reliable conclusions and there was a susceptibility testing for them in all four semesters. The wards that were included in this comparative study besides ICU were selected upon the adequate frequency of isolates in all four semesters.

### 4.4. Statistical Analysis

The overall 2-year and semester resistance rates of *P. aeruginosa*, *A. baumannii* and *K. pneumoniae* clinical isolates against individual antimicrobial agents were analyzed. Differences between means regarding the resistance of the three bacteria mentioned above between ICU and other wards were determined by univariate analysis of variance and Tukey’s multiple range tests. The relevant data were analyzed by the two years and by each semester separately. A significance level of *p* < 0.05 was used.

## 5. Conclusions

An increased antimicrobial resistance among Gram-negative bacteria was found in the ICU setting. This rise in Gram-negative isolates has serious health and economic consequences for the national healthcare system, leading to increased morbidity, prolonged length of hospitalization and fatalities. Comprehensive surveillance programs are needed to track the origin and emergence pathways of resistant pathogens. Treatment requires careful consideration due to limited options. Prompt antibiotic utilization and the development of multiple preventive strategies in conjunction with infection controls can optimize patient outcomes. A further survey of resistance profiles in certain antibiotics is required in order to measure the effectiveness of the surveillance programs implemented in our hospital.

## Appendix A

**Table A1:** Number of tests processed in different antibiotics, number of isolates collected, overall resistance per ward and semester and mean difference of resistance versus intensive care units (ICUs) regarding *P. aeruginosa* during the 2 years (2017–2018).

*P. aeruginosa*	Int. Med. A	Pulm. A	Int. Med. B	Pulm. B	Cystic	Urol.	ICU
Number of tests	1385	1939	1659	2120	10,711	1383	2639
Number of isolates	128	160	148	174	826	124	224
Analysis per Semester							
2017 (A)	30.56%	26.89%	31.28%	30.23%	42.80%	29.02%	41.94%
2017 (B)	29.93%	38.78%	38.25%	29.85%	32.88%	24.93%	47.44%
2018 (A)	13.07%	27.92%	21.27%	20.94%	36.91%	30.04%	53.11%
2018 (B)	24.91%	25.97%	32.14%	18.91%	35.74%	31.72%	50.66%
Overall resistance	25.70%	29.91%	32.67%	25.80%	37.10%	28.99%	49.03%
Mean difference of resistance vs. ICU	23.33% *	19.12% *	16.36% *	23.23% *	11.93% *	20.04% *	

* *p*-value is less than 0.05.

**Table A2:** Resistance rates for *P. aeruginosa* per ward per antibiotic during the 2 years (2017–2018).

*P. aeruginosa*	Int. Med. A	Pulm. A	Int. Med. B	Pulm. B	Cystic	Urol.	ICU
Amikacin	22.58%	29.68%	30.99%	19.53%	49.69%	35.59%	49.29%
Aztreonam	38.21%	43.33%	38.85%	43.03%	40.81%	42.74%	50.46%
Cefepime	31.62%	37.66%	34.56%	30.36%	46.31%	31.93%	56.67%
Ceftazidime	24.19%	30.00%	28.47%	26.51%	31.93%	14.88%	50.68%
Ciprofloxacin	28.81%	36.24%	43.17%	31.52%	32.78%	46.22%	53.23%
Colistin	0.00%	1.32%	2.86%	0.61%	6.03%	0.00%	2.59%
Doripenem	36.73%	27.35%	37.18%	24.11%	32.21%	21.21%	53.25%
Gentamicin	33.33%	34.64%	40.00%	31.74%	57.54%	41.88%	57.97%
Imipenem	25.98%	31.41%	36.62%	27.11%	39.14%	28.23%	54.76%
Levofloxacin	-	28.35%	55.32%	29.25%	35.98%	-	55.11%
Meropenem	27.78%	29.68%	34.51%	25.45%	32.02%	28.10%	55.02%
Piperacillin/tazobactam	23.02%	29.49%	25.35%	25.88%	30.46%	22.76%	49.06%
Tobramycin	22.61%	29.03%	34.59%	20.48%	32.45%	32.48%	47.55%

**Table A3:** Mean differences in resistance rates for *P. aeruginosa* in ICUs versus other wards per antibiotic during the 2 years (2017–2018).

*P. aeruginosa*	Int. Med. A	Pulm. A	Int. Med B	Pulm. B	Cystic	Urol.
Amikacin	26.71% *	19.61% *	18.30% *	29.76% *	−0.40%	13.70%
Aztreonam	12.25%	7.13%	11.61%	7.43%	9.65%	7.73%
Cefepime	25.04% *	19.00% *	22.11% *	26.31% *	10.36%	24.73% *
Ceftazidime	26.49% *	20.68% *	22.21% *	24.17% *	18.74% *	35.80% *
Ciprofloxacin	24.42% *	16.99% *	10.07%	21.72% *	20.45% *	7.02%
Colistin	2.59%	1.27%	−0.27%	1.98%	−3.44%	2.59%
Doripenem	16.52%	25.90% *	16.07%	29.14% *	21.05% *	32.04% *
Gentamicin	24.64% *	23.33% *	17.97% *	26.23% *	0.43%	16.09%
Imipenem	28.78% *	23.35% *	18.14% *	27.65% *	15.63% *	26.54% *
Levofloxacin	-	26.77% *	−0.21%	25.86% *	19.13% *	-
Meropenem	27.25% *	25.35% *	20.52% *	29.57% *	23.01% *	26.92% *
Piperacillin/tazobactam	26.04% *	19.57% *	23.70% *	23.17% *	18.60% *	26.29% *
Tobramycin	24.94% *	18.52% *	12.96%	27.07% *	15.10% *	15.07%

* *p*-value is less than 0.05.

**Table A4:** Number of tests processed in different antibiotics, number of isolates collected, overall resistance per ward and semester and mean difference of resistance versus ICUs regarding *A. baumannii* during the 2 years (2017–2018).

*A. baumannii*	Int. Med. A	Pulm. A	Pulm. B	ICU
Number of tests	905	1201	887	2616
Number of isolates	73	89	67	195
Analysis per semester				
2017 (A)	87.46%	89.41%	90.55%	89.32%
2017 (B)	88.26%	80.82%	66.82%	93.91%
2018 (A)	84.74%	84.58%	86.42%	95.16%
2018 (B)	89.00%	78.54%	75.38%	93.44%
Overall resistance	87.29%	84.76%	81.29%	93.00%
Mean difference of resistance vs. ICU	5.71% *	8.24% *	11.72% *	

* *p*-value is less than 0.05.

**Table A5:** Resistance rates for *A. baumannii* per ward per antibiotic during the 2 years (2017–2018).

*A. baumannii*	Int. Med. A	Pulm. A	Pulm. B	ICU
Amikacin	90.00%	89.77%	80.00%	97.27%
Ampicillin/sulbactam	94.20%	88.64%	87.88%	96.79%
Cefepime	94.20%	89.66%	85.94%	98.89%
Cefotaxime	98.28%	90.70%	90.63%	99.43%
Ceftazidime	97.14%	89.77%	89.23%	98.37%
Ciprofloxacin	94.92%	90.80%	87.30%	98.89%
Colistin	30.56%	26.14%	37.50%	53.37%
Gentamicin	95.59%	88.24%	82.54%	98.33%
Imipenem	94.74%	93.83%	91.84%	100.00%
Levofloxacin	97.06%	90.24%	83.08%	98.84%
Meropenem	92.86%	89.66%	84.62%	98.91%
Minocycline	83.33%	86.25%	72.31%	84.27%
Tobramycin	80.82%	86.05%	79.69%	94.57%
TMP/SMX	90.00%	88.64%	87.69%	90.53%

**Table A6:** Mean differences of resistance rates for *A. baumannii* in ICUs versus other wards per antibiotic during the 2 years (2017–2018).

*A. baumannii*	Int. Med. A	Pulm. A	Pulm. B
Amikacin	7.27%	7.50%	17.27% *
Ampicillin/sulbactam	2.59%	8.16%	8.91%
Cefepime	4.69%	9.23% *	12.95% *
Cefotaxime	1.15%	8.73% *	8.80% *
Ceftazidime	1.23%	8.60% *	9.14% *
Ciprofloxacin	3.97%	8.08% *	11.59% *
Colistin	22.81% *	27.23% *	15.87%
Gentamicin	2.75%	10.10% *	15.79% *
Imipenem	5.26%	6.17% *	8.16% *
Levofloxacin	1.78%	8.59% *	15.76% *
Meropenem	6.05%	9.25% *	14.29% *
Minocycline	0.94%	−1.98%	11.96%
Tobramycin	13.74% *	8.52%	14.88% *
TMP/SMX	0.53%	1.89%	2.83%

* *p*-value is less than 0.05.

**Table A7:** Number of tests processed in different antibiotics, number of isolates, overall resistance per ward and semester and mean difference of resistance versus ICUs regarding *K. pneumoniae* during the 2 years (2017–2018).

*K. pneumoniae*	Int. Med. A	Pulm. A	Int. Med. B	Pulm. B	Urol.	ICU
Number of tests	2109	2286	2702	2054	2114	3653
Number of isolates	133	135	164	117	125	204
Analysis per semester						
2017 (A)	48.46%	49.41%	59.14%	49.90%	32.48%	80.00%
2017 (B)	51.26%	44.84%	46.89%	46.85%	43.65%	68.45%
2018 (A)	40.40%	38.14%	45.70%	35.55%	45.04%	69.50%
2018 (B)	41.41%	44.88%	51.46%	35.57%	40.07%	72.06%
Overall resistance	46.23%	43.96%	52.48%	42.06%	40.78%	72.30%
Mean difference of resistance vs. ICU	26.07% *	28.33% *	19.82% *	30.23% *	31.52% *	-

* *p*-value is less than 0.05.

**Table A8:** Resistance rates for *K. pneumoniae* per ward per antibiotic during the 2 years (2017–2018).

*K. pneumoniae*	Int. Med. A	Pulm. A	Int. Med. B	Pulm. B	ICU	Urol.
Amikacin	34.38%	27.07%	33.75%	30.77%	60.78%	27.27%
Amoxicillin/clavulanic acid	57.60%	56.15%	64.83%	54.05%	83.98%	49.17%
Ampicillin/sulbactam	65.66%	62.24%	73.81%	60.22%	85.98%	58.70%
Cefepime	54.64%	55.56%	61.54%	50.00%	81.29%	48.91%
Cefotaxime	52.43%	54.55%	61.48%	48.51%	81.61%	45.92%
Cefoxitin	43.44%	40.31%	50.98%	42.86%	78.84%	35.90%
Ceftazidime	49.19%	51.54%	58.97%	45.61%	80.21%	42.15%
Cefuroxime	53.54%	57.14%	66.24%	51.33%	81.72%	52.46%
Ciprofloxacin	57.84%	51.92%	63.36%	49.50%	83.04%	53.26%
Colistin	14.78%	11.48%	12.12%	16.35%	39.49%	14.42%
Ertapenem	45.45%	40.40%	51.20%	43.96%	81.44%	39.13%
Gentamicin	32.20%	29.75%	41.22%	33.64%	54.21%	23.28%
Imipenem	37.82%	37.60%	46.05%	38.18%	79.03%	30.17%
Levofloxacin	-	40.48%	55.38%	38.20%	79.04%	-
Meropenem	36.07%	36.64%	45.81%	36.28%	78.31%	29.75%
Moxifloxacin	-	50.00%	-	38.60%	82.58%	-
Piperacillin/tazobactam	47.58%	42.64%	50.63%	45.95%	80.75%	39.84%
Tetracycline	30.00%	40.19%	39.37%	26.92%	29.47%	37.50%
Tigecycline	-	-	-	-	3.57%	-
Tobramycin	56.73%	50.46%	59.26%	48.98%	80.45%	46.00%
Trimethoprim/sulfamethoxazole	54.26%	51.91%	55.70%	42.98%	77.13%	50.40%

**Table A9:** Mean differences of resistance rates for *K. pneumoniae* in ICUs versus other wards per antibiotic during the 2 years (2017–2018).

*K. pneumoniae*	Int. Med. A	Pulm. A	Int. Med. B	Pulm. B	Urol.
Amikacin	26.41% *	33.72% *	27.03% *	30.02% *	33.51% *
Amoxicillin/clavulanic acid	26.38% *	27.82% *	19.15% *	29.92% *	34.81% *
Ampicillin/sulbactam	20.32% *	23.73% *	12.17%	25.76% *	27.28% *
Cefepime	26.65% *	25.73% *	19.75% *	31.29% *	32.37% *
Cefotaxime	29.18% *	27.06% *	20.13% *	33.09% *	35.69% *
Cefoxitin	35.39% *	38.53% *	27.86% *	35.98% *	42.94% *
Ceftazidime	31.02% *	28.68% *	21.24% *	34.60% *	38.07% *
Cefuroxime	28.18% *	24.58% *	15.48% *	30.39% *	29.26% *
Ciprofloxacin	25.20% *	31.12% *	19.68% *	33.54% *	29.78% *
Colistin	24.70% *	28.01% *	27.37% *	23.14% *	25.06% *
Ertapenem	35.98% *	41.03% *	30.24% *	37.48% *	42.31% *
Gentamicin	22.01% *	24.46% *	12.99%	20.57% *	30.93% *
Imipenem	41.22% *	41.43% *	32.98% *	40.85% *	48.86% *
Levofloxacin	-	38.57% *	23.66% *	40.84% *	-
Meropenem	42.24% *	41.67% *	32.50% *	42.02% *	48.55% *
Moxifloxacin	-	32.58% *	-	43.98% *	-
Piperacillin/tazobactam	33.17% *	38.11% *	30.12% *	34.80% *	40.91% *
Tetracycline	−0.53%	−10.71%	−9.90%	2.55%	−8.03%
Tobramycin	23.72% *	29.99% *	21.19% *	31.47% *	34.45% *
Trimethoprim/sulfamethoxazole	22.86% *	25.22% *	21.43% *	34.15% *	26.73% *

* *p*-value is less than 0.05.
